# Dual Culprit ST-Elevation Myocardial Infarction: A Rare but Complex Clinical Entity

**DOI:** 10.7759/cureus.90478

**Published:** 2025-08-19

**Authors:** Abhinav Karan, Julien Feghaly, Anamarys Blanco, Calvin Choi

**Affiliations:** 1 Internal Medicine, University of Florida College of Medicine – Jacksonville, Jacksonville, USA; 2 Cardiovascular Diseases, University of Florida College of Medicine – Jacksonville, Jacksonville, USA; 3 Cardiology, University of Florida College of Medicine – Jacksonville, Jacksonville, USA; 4 Interventional Cardiology, University of Florida College of Medicine – Jacksonville, Jacksonville, USA

**Keywords:** acute coronary syndrome, anginal chest pain, dual culprit, dual culprit stemi, heart failure with reduced ejection fraction, significant coronary artery disease, st-elevation myocardial infarction (stemi)

## Abstract

Dual culprit ST-elevation myocardial infarction (STEMI) refers to the simultaneous involvement of two different coronary arteries, both of which contribute to the ischemic event. It is distinct from multiple plaque ruptures in a single vessel and poses additional challenges, especially regarding timely and effective intervention. Dual culprit STEMI presents several unique diagnostic and management challenges that differentiate it from traditional single-vessel STEMI. The simultaneous involvement of two different coronary artery territories can lead to an atypical presentation and complicate clinical decision-making. Diagnosing dual culprit STEMI requires a high degree of clinical suspicion and thorough imaging. Electrocardiogram (ECG) findings may be more diffuse and nonspecific than in single-vessel STEMI, as ischemic changes may be observed in multiple coronary artery territories. Coronary angiography remains the gold standard for diagnosis. However, the challenge lies in identifying both culprit lesions during the acute phase of the STEMI. The management of dual culprit STEMI poses unique therapeutic challenges, particularly in deciding which vessel to treat first, and how to manage the other lesion(s) within the constraints of the acute setting. Dual culprit STEMI carries a higher risk of adverse outcomes compared to single-vessel STEMI. The presence of two culprit lesions is associated with a larger area of myocardial ischemia, which can lead to higher rates of heart failure, arrhythmias, and mortality. Here, we discuss two cases of dual-culprit STEMI and an approach to diagnosis and management.

## Introduction

ST-elevation myocardial infarction (STEMI) is most commonly caused by a sudden occlusion of a coronary artery due to plaque rupture or erosion, leading to myocardial ischemia and necrosis. In the majority of cases, a single culprit lesion is identified as the cause of the acute event. However, in rare cases, two distinct coronary artery lesions may be responsible for a single STEMI event. This phenomenon, known as "dual culprit STEMI," presents a diagnostic and therapeutic challenge for clinicians due to its atypical presentation and complexity [1].

Dual culprit STEMI refers to the simultaneous involvement of two different coronary arteries, both contributing to the ischemic event. The incidence of dual culprit STEMI is low, accounting for 2-3% of all STEMI cases in some studies, though this is likely a clinical underestimate [2]. Differentiating between one extensive culprit lesion causing widespread ischemia and two simultaneous culprit lesions can be difficult, and one lesion may overshadow another in clinical assessment. In addition, standard angiography without advanced imaging modalities such as intravascular ultrasound or optical coherence tomography may underestimate unstable plaque and plaque rupture [2]. Despite this, its clinical importance is high, given its potential for misdiagnosis, delayed treatment, and poor outcomes if not promptly recognized and treated due to incomplete revascularization with delayed treatment and larger infarct size [1].

Dual culprit STEMI typically results from a complex interplay between underlying cardiovascular risk factors, such as diabetes mellitus, hypercoagulable states, multivessel coronary artery disease, and systemic inflammatory conditions, and is associated with extensive myocardial injury and poor clinical outcomes [3]. Management is challenging and requires prompt diagnosis, rapid revascularization, and meticulous supportive care to mitigate complications such as cardiogenic shock, acute heart failure, and fatal arrhythmias [1].

In this article, we discuss the pathophysiology, diagnostic approach, and management considerations for dual culprit STEMI, using two cases to highlight the unique challenges faced in this rare but important condition.

## Case presentation

Case 1

A 63-year-old male with a prior history of hypertension, hyperlipidemia, tobacco use disorder, and a prior non-ST-segment elevation myocardial infarction (NSTEMI) with percutaneous coronary intervention (PCI) to the mid-right coronary artery (RCA) presented to the emergency room with the acute onset of chest pain for the past two hours before presentation. He noted that the pain was similar to his prior NSTEMI. He had been non-compliant with his medications, including aspirin and atorvastatin. His initial vital signs were significant for a blood pressure of 142/99 mmHg, with a heart rate of 89 bpm and oxygen saturation (SpO2) of 96% on room air.

His initial EKG revealed ST elevations in leads II, III, and aVF, with reciprocal ST depressions in leads V1-V4. His echocardiogram at bedside revealed a newly severely reduced left ventricular ejection fraction (LVEF) at 35%, with basal inferior segment akinesis and multiple other segments being hypokinetic.

**Figure 1:**
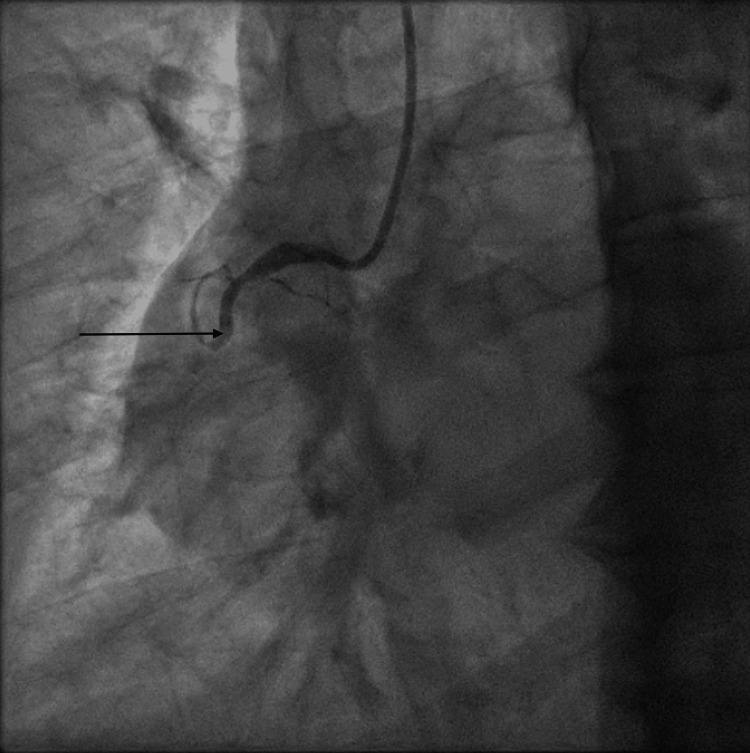
Coronary angiogram of the right system showing acute 100% thrombotic occlusion of the right coronary artery. Black arrow: Acute thrombotic occlusion of the right coronary artery with no distal flow.

**Figure 2:**
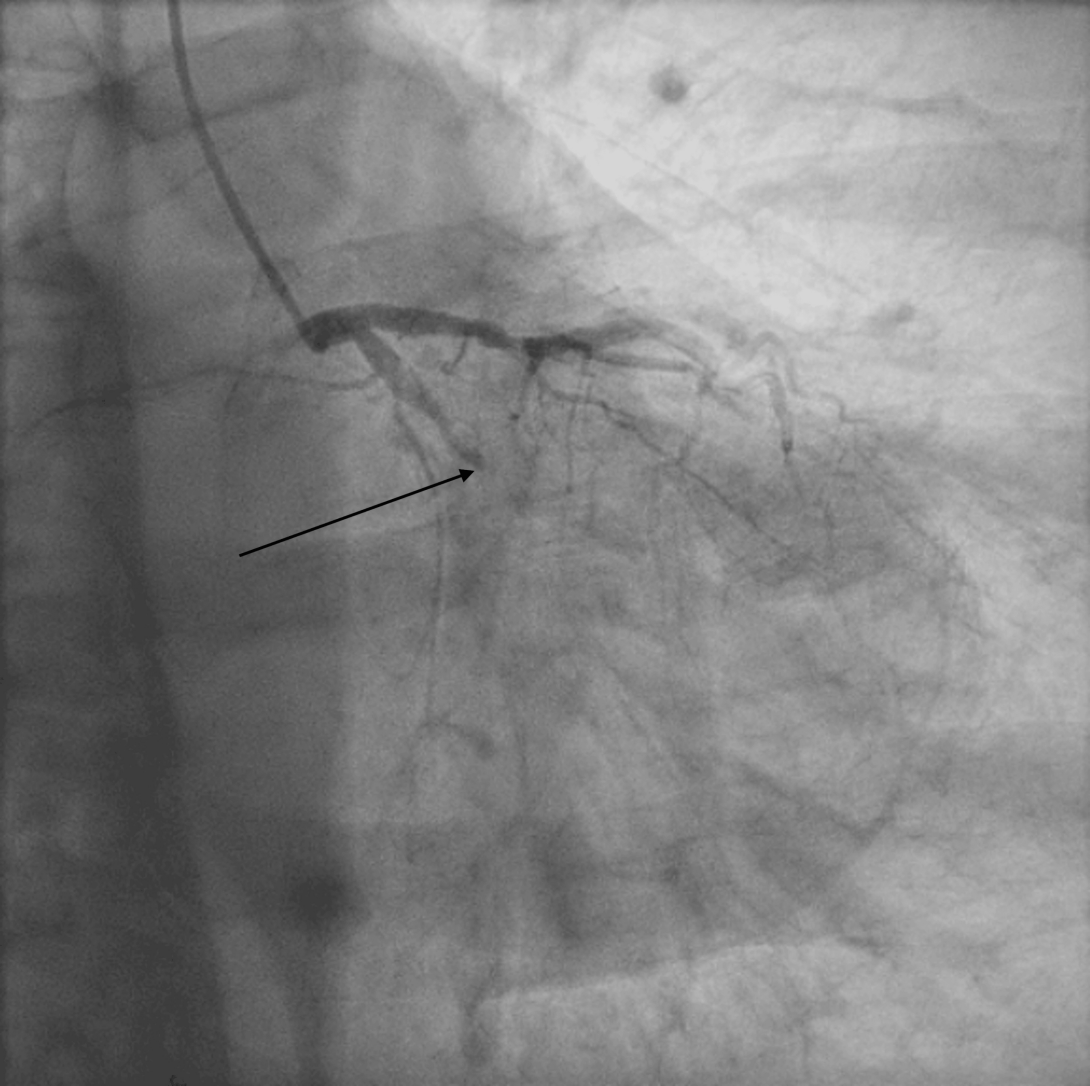
Coronary angiogram of the left system showing acute thrombotic occlusion of the obtuse marginal branch 1 of the left circumflex artery. Black arrow: Acute thrombotic occlusion of the obtuse marginal branch 1 of the left circumflex artery.

He was transferred immediately to the cardiac catheterization lab for emergent angiography. Angiography revealed an acute thrombotic occlusion of both the proximal RCA (Figure 1 and Video 1), as well as the proximal obtuse marginal branch 1 (OM1) (Figure 2 and Video 2), both considered possible culprit lesions due to Thrombolysis in Myocardial Infarction (TIMI) 0 flow across the vessel.

**Video 1:** Acute thrombotic occlusion of the proximal right coronary artery.

**Video 2:** Acute thrombotic occlusion of the obtuse marginal branch 1.

**Figure 3:**
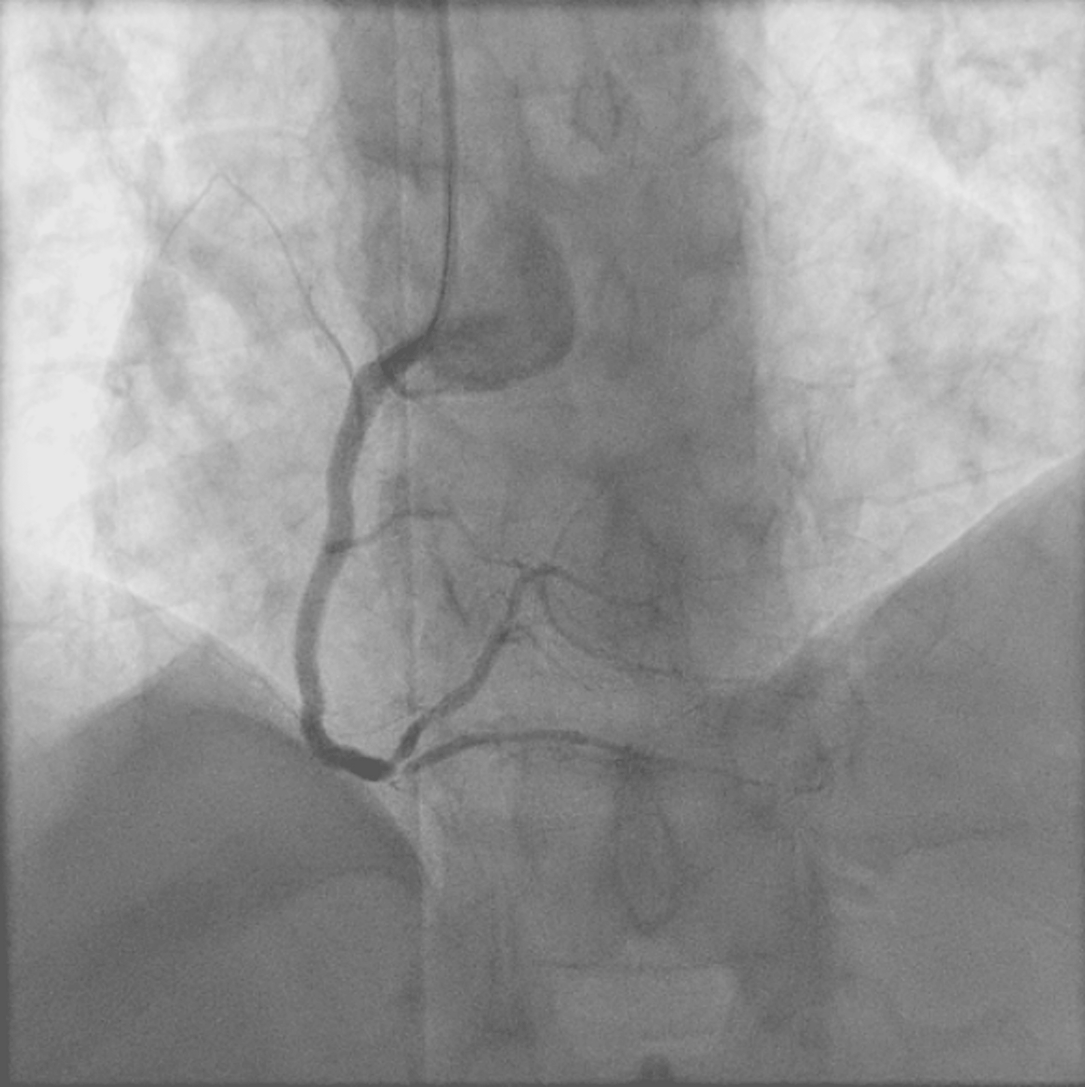
Coronary angiogram of the right system showing revascularized right coronary artery following percutaneous coronary intervention.

**Figure 4:**
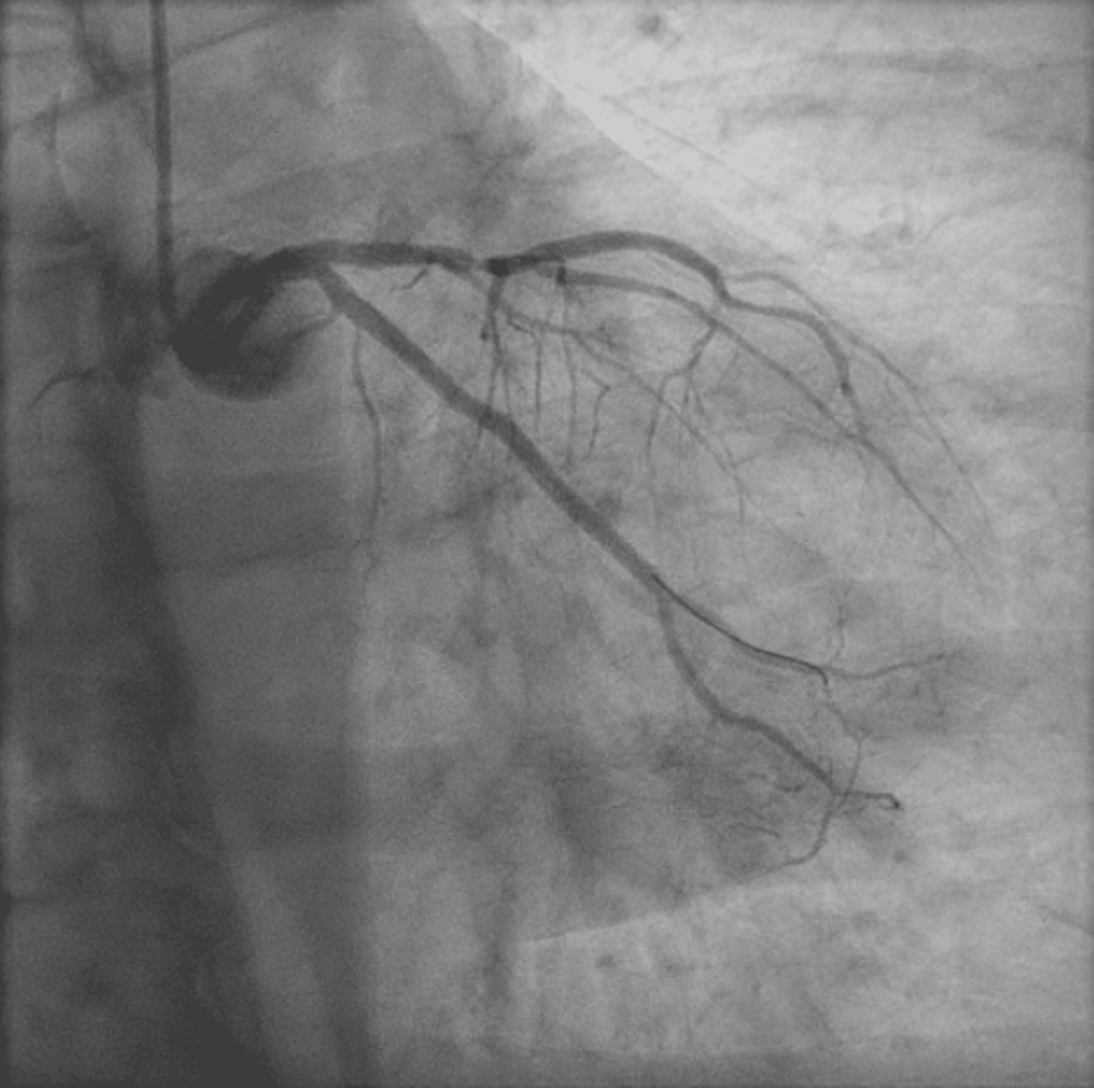
Coronary angiogram of the left system showing revascularized obtuse marginal branch 1 following percutaneous coronary intervention.

The patient subsequently underwent PCI to the proximal-distal RCA across the prior mid-RCA stent using a 3.0 x 48 mm Synergy drug eluting stent (DES) distally, and a 3.5 x 38 mm Onyx Frontier DES proximally, and then PCI of the proximal to mid OM1 using a 2.5 x 48 mm Synergy DES (Figures 3, 4 and Videos 3, 4), with reported resolution of chest pain and normalization of ST segment changes on continuous electrocardiographic monitoring during revascularization. The patient was also noted to have 60% occlusion of the mid left anterior descending artery (LAD), which was planned for staged revascularization at a later date due to risk of contrast nephropathy. The patient was loaded with prasugrel 60 mg qd and was counseled to continue dual antiplatelet therapy (DAPT) with prasugrel 10 mg daily and acetylsalicylic acid (ASA) 81 mg daily, given his high thrombotic risk.

**Video 3:** Coronary angiogram of revascularized right coronary artery following percutaneous coronary intervention.

**Video 4:** Coronary angiogram of revascularized obtuse marginal branch 1 following percutaneous coronary intervention.

Case 2

A 74-year-old male with a prior history of hypertension, coronary artery disease with a prior PCI to the left circumflex artery and mid-LAD, and hyperlipidemia presented for the acute onset of chest pain for the past three hours. Initial vital signs were significant for a blood pressure of 129/79 mmHg, a heart rate of 86 bpm, and SpO2 of 100%. An EKG revealed ST elevation in leads I, aVL, V5, and V6. Transthoracic echocardiography (TTE) revealed a newly severely reduced LVEF of 20-25% with akinesis of the basal anteroseptal wall, and hypokinesis of the mid and distal basal segments.

**Figure 5:**
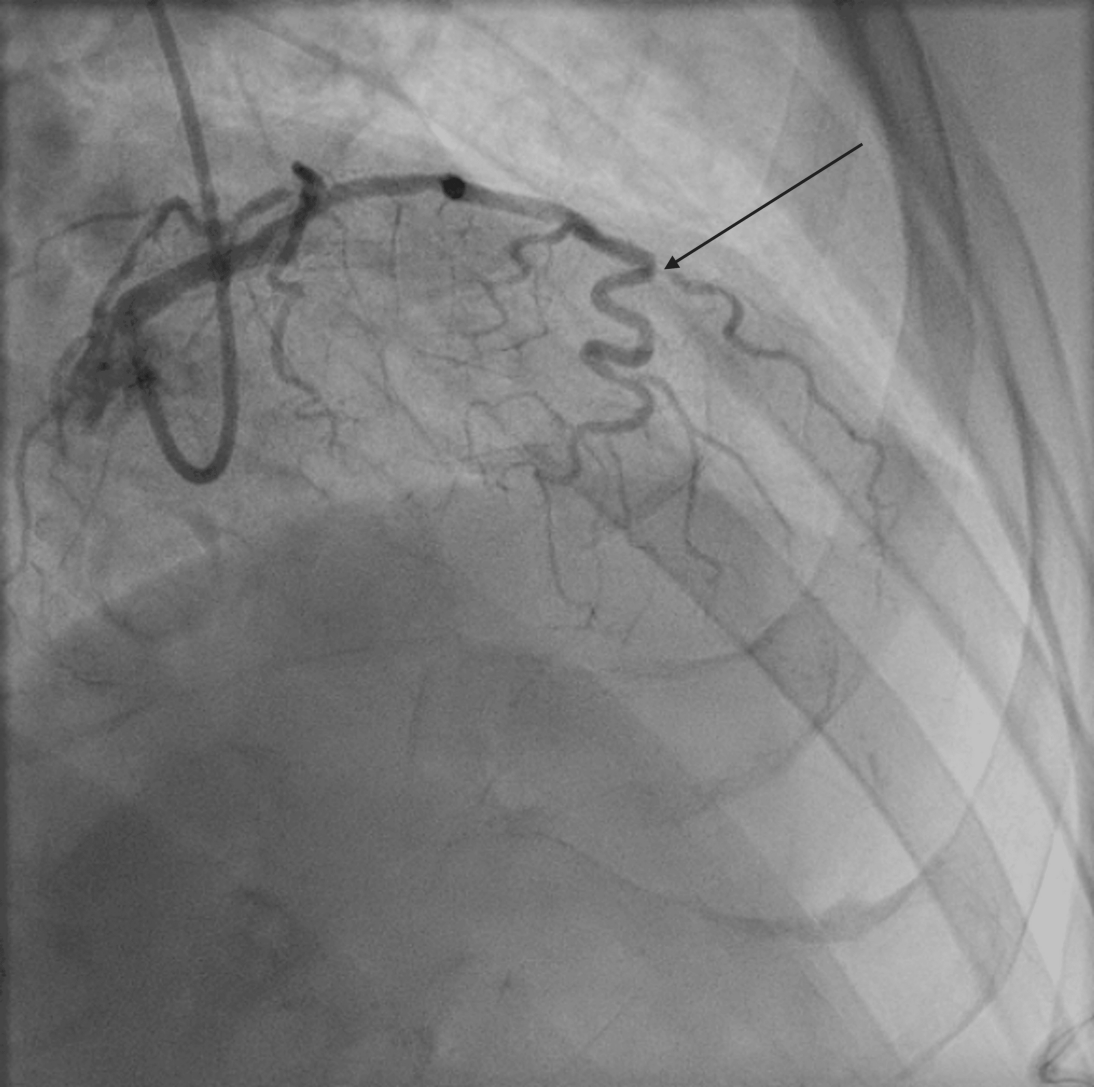
Coronary angiogram of the left system, revealing hazy, acute thrombotic occlusion of the left anterior descending artery. Black arrow: Hazy, acute thrombotic occlusion of the left anterior descending artery.

**Figure 6:**
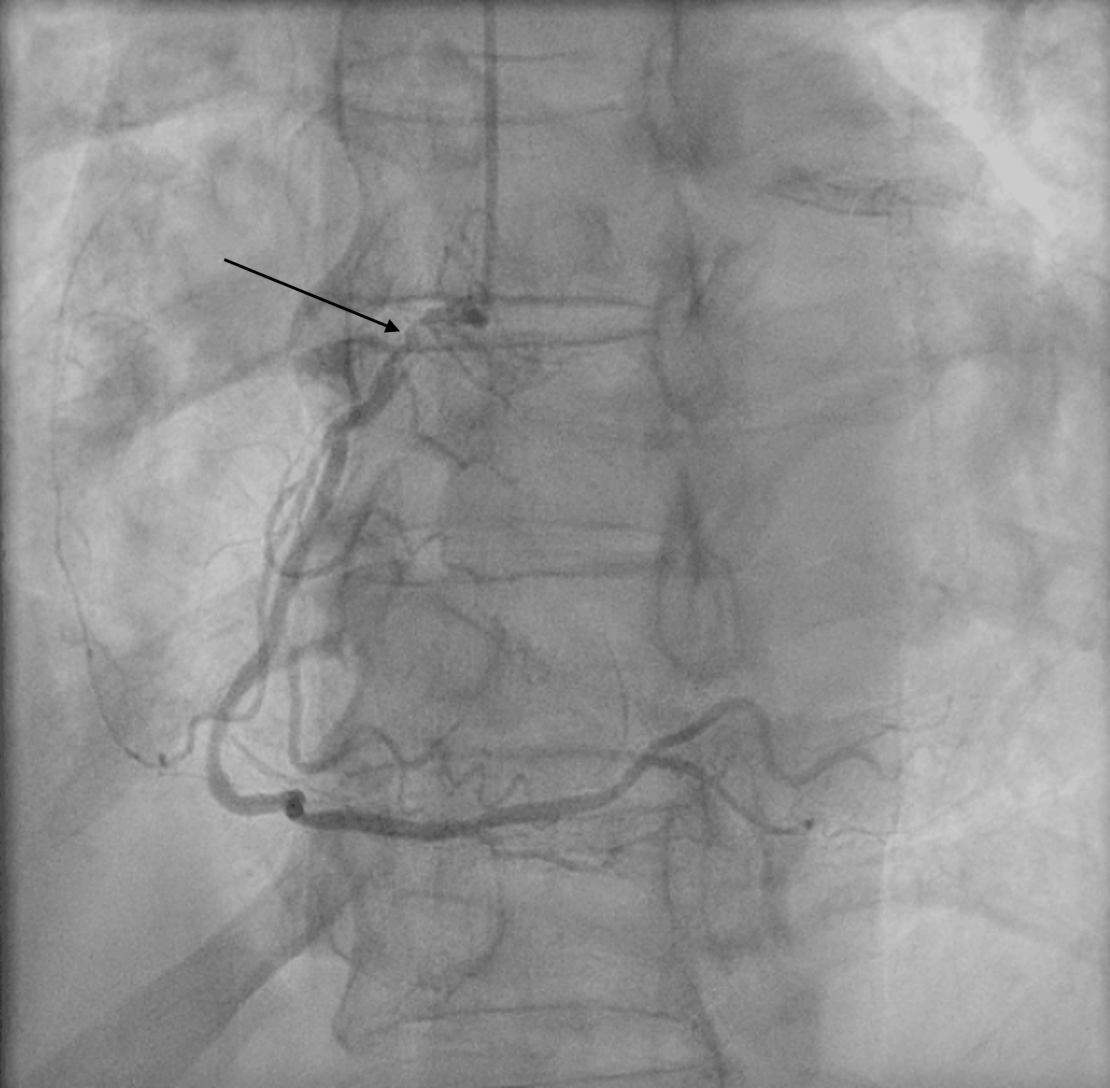
Coronary angiogram showing acute thrombotic occlusion of the proximal right coronary artery. Black arrow: Acute thrombotic occlusion of the proximal right coronary artery.

Emergent cardiac catheterization revealed an acute 100% in-stent thrombotic occlusion of the proximal LAD (Figure 5 and Video 5), as well as an acute 99% thrombotic occlusion of the proximal RCA (Figure 6 and Video 6), both considered culprit lesions.

**Video 5:** Coronary angiogram of the left system with acute thrombotic occlusion of the left anterior descending artery.

**Video 6:** Coronary angiogram of the right coronary artery with acute thrombotic occlusion of the proximal segment.

**Figure 7:**
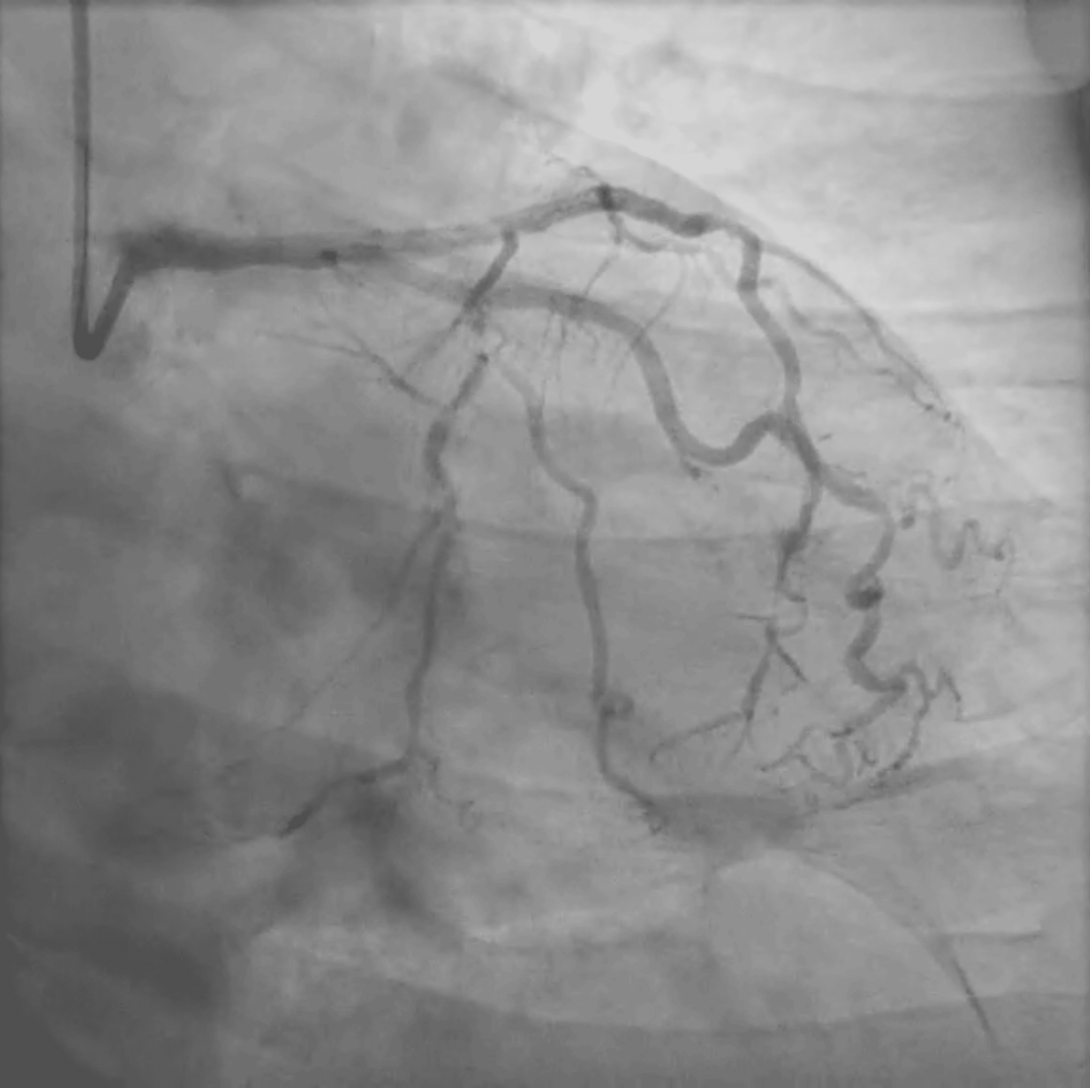
Coronary angiogram of the left system with revascularized left anterior descending artery following percutaneous coronary intervention.

**Figure 8:**
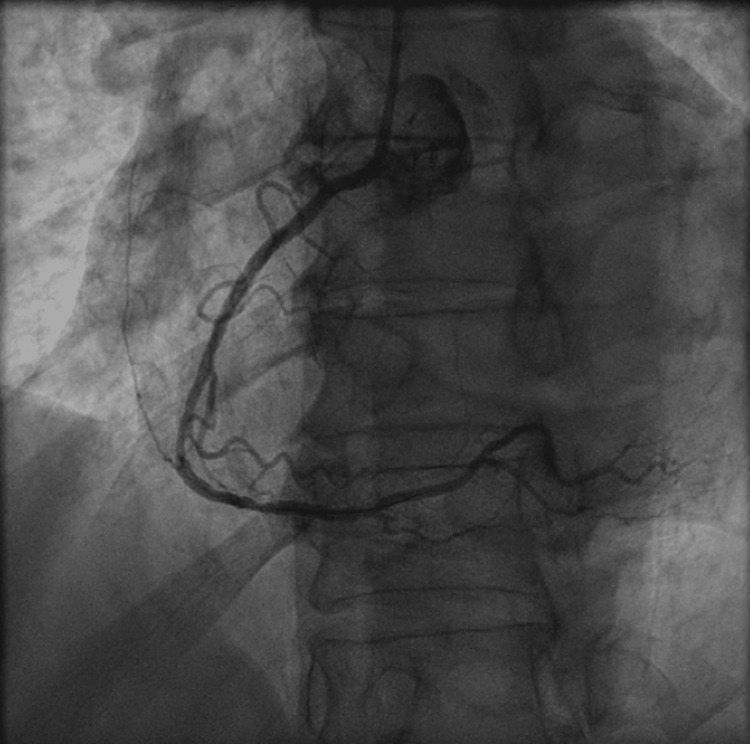
Coronary angiogram of revascularized right coronary artery following percutaneous coronary intervention.

The patient underwent successful PCI of the mid LAD in an overlapping fashion with a prior stent with a 2.5 x 22 mm Onyx DES (Figure 7) and PCI to the ostial-proximal RCA with a 3.0 x 30 mm Onyx DES (Figure 8). The patient was loaded with ticagrelor 180 mg and continued on DAPT with aspirin and ticagrelor thereafter.

## Discussion

Dual culprit STEMI poses distinct diagnostic and therapeutic challenges compared to conventional single-vessel STEMI. The concurrent occlusion of two separate coronary arteries may result in an unusual clinical presentation, making diagnosis and treatment more complex.

The incidence of dual culprit STEMI remains a critical epidemiological metric for interventional cardiologists, with current evidence suggesting significant underrecognition in clinical practice. The estimated angiographically confirmed incidence of dual culprit STEMI is roughly 2.5% among PCI-treated STEMI populations, though this is likely an underestimate of the true incidence [1]. Distinguishing between a single large culprit lesion responsible for extensive ischemia and two concurrent culprit lesions can be challenging, as one lesion may overshadow the other during clinical evaluation. Moreover, conventional angiography alone often fails to fully detect unstable plaques and ruptures without the aid of advanced imaging techniques like intravascular ultrasound (IVUS) or optical coherence tomography (OCT) [2].

The pathophysiology of dual culprit STEMI is complex and not fully understood. One theory suggests that the simultaneous rupture or erosion of plaques in different coronary arteries may occur due to a systemic inflammatory or prothrombotic state. Analysis of 47 dual-culprit STEMI cases revealed 62% lacked traditional triggers, suggesting diffuse endothelial activation from conditions like COVID-19-associated hypercoagulability, malignancy, or autoimmune disorders [1]. The intense inflammatory response, or cytokine storm, in conditions such as those aforementioned, leads to increased platelet activation and aggregation, raising the risk of multiple thrombus formations simultaneously in different coronary arteries [3]. Furthermore, studies have shown that patients with dual culprit lesions often have higher inflammatory markers, such as an elevated C-reactive protein level and elevated interleukin-6 levels greater than 50 pg/mL, compared to those with single-vessel STEMI [2]. Additionally, conditions such as hyperlipidemia, diabetes, and smoking have been identified as common risk factors that may predispose individuals to such events [3]. Another hypothesis involves the possibility of coronary spasm or microvascular dysfunction affecting multiple vessels concurrently, though this is less commonly reported [4].

Diagnosing dual culprit STEMI requires a high degree of clinical suspicion and thorough imaging. ECG findings may be more diffuse and nonspecific than in single-vessel STEMI, as ischemic changes may be observed in multiple coronary artery territories. This can make it difficult to localize the culprit lesion based on the ECG alone. Up to 68% of patients with dual culprit STEMI present with inferior ST elevations, and some features found to increase pre-test probability for multi-vessel culprit lesions in observational studies are as follows: (1) ST depression > eight leads; (2) aVR ST elevation > 1 mm with prolonged QRS; (3) dynamic QTc prolongation to >500 ms [2-6].

Coronary angiography remains the gold standard for diagnosis. However, the challenge lies in identifying both culprit lesions during the acute phase of the STEMI. In some cases, one lesion may be more obvious, leading to the false assumption that only a single vessel is involved. Intracoronary imaging techniques, such as OCT or IVUS, can help differentiate between culprit and non-culprit lesions by providing detailed plaque morphology, though these techniques are not always readily available in the acute setting [6].

The management of dual culprit STEMI poses unique therapeutic challenges, particularly in deciding which vessel to treat first and how to manage the other lesion(s) within the constraints of the acute setting. There is no universal consensus or guideline statement on dual culprit STEMI. It is reasonable to first treat the most hemodynamically significant lesion or the artery supplying the largest myocardial territory with primary PCI. This is often followed by staged PCI of the second culprit lesion during the same or a subsequent procedure, depending on the patient’s stability and clinical condition [5,6].

Dual culprit STEMI carries a higher risk of adverse outcomes compared to single-vessel STEMI. The presence of two culprit lesions is associated with a larger area of myocardial ischemia, which can lead to higher rates of heart failure, arrhythmias, and mortality [4]. Early recognition and appropriate treatment of both lesions are critical to improving outcomes in these patients. Furthermore, due to time constraints in the acute setting of a STEMI, advanced imaging strategies such as IVUS and OCT may become time-consuming and limit door-to-balloon time.

Despite advances in interventional cardiology, the prognosis of dual culprit STEMI remains worse than that of single-vessel STEMI. Several studies have shown that patients with dual culprit lesions are more likely to experience recurrent myocardial infarction, repeat revascularization, and higher mortality at follow-up [7]. Therefore, early identification and aggressive management are essential in reducing the risk of adverse outcomes.

## Conclusions

Dual culprit STEMI is a rare and complex clinical entity that requires careful consideration in both diagnosis and management. The simultaneous involvement of two coronary arteries complicates the acute care of these patients, with higher risks for misdiagnosis and adverse outcomes if not promptly addressed. As interventional cardiology continues to evolve, enhanced imaging techniques and a multidisciplinary approach will likely play a key role in improving the prognosis of patients with dual culprit STEMI. Further research is needed to better understand the underlying mechanisms and to develop more effective treatment strategies for this challenging condition.
